# Zebras in the Era of COVID

**DOI:** 10.7759/cureus.31863

**Published:** 2022-11-24

**Authors:** Parker Foster, Kathryn Golab, Ajaya Sharma, Hari R Paudel

**Affiliations:** 1 Internal Medicine, Medical College of Wisconsin, Milwaukee, USA; 2 Hematology, Wisconsin Diagnostic Laboratories, Milwaukee, USA

**Keywords:** infectious disease pathology, interprofessional education and collaboration, public health problems, malaria treatment, falciparum malaria

## Abstract

Malaria remains a significant global health concern. Non-malarial areas, including North America and Europe, largely report cases in association with recent travel to endemic regions. Though cyclic symptoms and chills are characteristic of the infection, thorough social histories including previous travels is the basic prerequisite for timely diagnosis, treatment initiation, and ultimate prognosis of this potentially life-threatening condition.

## Introduction

Despite highly effective treatments, malaria remains a significant global health concern, accounting for a significant share of the morbidity and mortality in the developing world [1]. Transmitted by the female Anopheles mosquito, malaria is caused by one of five known species of the protozoan Plasmodium, including *P. falciparum*, *P. vivax*, *P. ovale*, *P. malariae*, and *P. knowlesi*. Infection with *P. falciparum* is responsible for the most severe and fatal forms, causing 90% of the world’s malaria mortality [2]. Notably, each species demonstrates specific qualities that influence its presentation and treatment considerations. For example, while the incubation period can range from seven to 30 days for each species, *P. falciparum* has been observed to present on the shorter end of the spectrum. Importantly, the incubation time can be more prolonged in individuals with partial immunity or those taking incompletely effective prophylaxis, creating a significant gap between exposure and presentation [3]. Alternatively, both *P. vivax* and *P. ovale* may adopt a dormant phase, characterized by a persistence of hypnozoites in hepatocytes, creating the potential for late presentation or relapse [3, 4]. Treatment with agents such as primaquine is therefore used to eliminate lingering hypnozoites. Because *P. falciparum* has no exoerythrocytic phase, primaquine has no role in attaining a cure, and is thus generally not given. The World Health Organization has however recommended the addition of primaquine in the treatment regimen for *P. falciparum* in certain areas to aid in blocking transmission [5]. Finally, despite no exoerythrocytic phase, *P. malariae* has been reported to persist in the blood at very low densities in a state of dormancy, with a recrudescence possible decades later [6].

North America and Europe are considered non-malarial areas, with cases predominately attributable to travel within endemic regions [7]. Management of malaria is dependent on early recognition and treatment initiation. Here, we present a case of uncomplicated falciparum malaria in Milwaukee, Wisconsin.

## Case presentation

A 64-year-old Caucasian male with a previous medical history of self-reported malaria in 2003 and latent tuberculosis s/p isoniazid therapy, presented one day after a sudden onset of fever, chills, teeth chattering, diaphoresis, and malaise. The patient reported that the symptoms occur twice daily, and last for 1 hour. Between episodes, the patient endorsed fatigue, nausea, headaches, and poor appetite. The patient also noted a sensation of dizziness during flares, which corresponded with a drop in blood pressure to 80s systolic. Laboratory values on admission are noted in Table 1.

**Table 1:** Laboratory Panel and Studies. Only laboratory values outside the standard reference range were included. All other labs were within normal limits at the time of admission. *Reference range for creatinine was patient’s baseline.

Lab	Patient	Reference Range
Sodium	132mmol/L	136-145mmol/L
Bicarbonate	19mmol/L	22-29mmol/L
Blood Urea Nitrogen (BUN)	31mg/dL	6-23mg/dL
Creatinine	1.88mg/dL	0.9-1.0mg/dL*
Aspartate Transferase (AST)	73U/L	13-44U/L
Total Bilirubin	2.2mg/dL	0.2-1.2mg/dL
Hemoglobin	13.5g/dL	13.7-17.5g/dL
Platelets	53 x 10^3^/µL	150-450 x 10^3^/µL
Prothrombin Time (PT)	15.5sec	9.5-11.8sec
Partial Thromboplastin Time (PTT)	34.1sec	23-30sec

**Figure 1:**
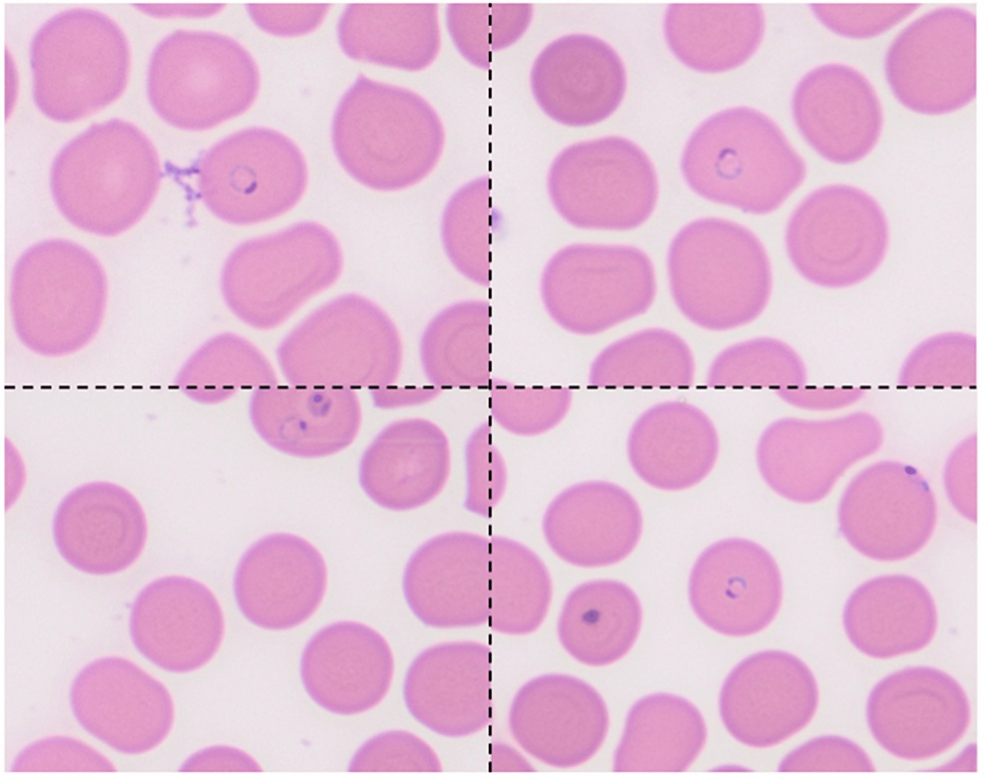
Blood Parasite Smear. Intraerythrocytic *Plasmodium falciparum* rings. Parasitemia of 3.6%. Prepared as thin prep slides stained with traditional Wright-Giemsa stain at 100x. Imaging spliced to highlight relevant findings, with dashed line to denote cutoffs.

The patient reported that symptoms were similar to a previous malarial episode in 2003. Social history revealed that the patient recently returned from Ghana, did not take antimalarial prophylaxis, and recalled a mosquito bite on the first night of his trip (15 days prior to symptom onset). A peripheral blood smear revealed intracellular organisms most consistent with plasmodium species, which was confirmed by serological antigen testing. The antigen testing was positive for both Plasmodium falciparum antigen and pan malarial antigen. Peripheral parasitemia peaked at 3.6% infected red blood cells (Figure 1). The patient was diagnosed with uncomplicated falciparum malaria and was started on artemisinin-based combination therapy (ACT), twice daily for three days, per Infectious Disease recommendations. Complete metabolic panel, blood count, and parasite smear were repeated every 6 hours, with plans to initiate Artesunate 2.4mg/kg IV if the patient progressed to severe malaria.

The patient reported that cyclic flares became less intense following Coartem initiation. The degree of parasitemia showed a persistent downtrend over the subsequent two days toward undetectable levels. Notably, creatinine showed an uptrend to a peak of 2.38mg/dL on day 2 of admission, before subsequently downtrending following continuous maintenance fluids (lactated ringers 100cc/hr). On day 3 of admission, the patient noted a cough with inspiration, describing it as originating from deep in his chest. Physical exam was significant for bibasilar crackles. Given the creatinine downtrend, fluids were discontinued, resulting in improvements in the patient's cough. The patient was discharged following the completion of Coartem dosing, with plans for outpatient laboratory testing and monitoring by infectious disease.

## Discussion

Malaria can be categorized into either uncomplicated or severe presentations. The World Health Organization has established specific criteria for severe malaria [8]. These criteria utilize numerous factors, including metabolic chemistries, blood counts, renal and pulmonary function, and the degree of parasitemia, to name a few. Notably, *P. falciparum* is overwhelmingly responsible for severe presentations when compared to alternative species [9]. Those who lack previous exposure to malarial parasites, as well as those who have lost their immunity by leaving endemic areas for prolonged periods, are at the highest risk for developing a severe presentation [10, 11]. Thus, until proven otherwise, malaria should be amongst the leading differentials in any patient presenting with a febrile illness in the setting of travel history to malarial endemic regions.

ACTs are currently considered the best treatment for uncomplicated falciparum malaria, given their rapid and reliable efficacy [12]. In contrast, chloroquine, once widely used for both prophylaxis and treatment, is now rarely used unless the sensitivity of the strain prevalent in the area is known. Chloroquine-resistant malaria has become significantly widespread [13], and concern for artemisinin-resistance has been rising [14]. In response to this problem, it has been reported that ACTs remain the mainstay of therapy, but with a prolonged course. Rather than the standard three-day course, utilizing a six-day course showed good efficacy, with an associated cure rate of 97.7% [14].

Though our patient only met the criteria for uncomplicated falciparum malaria, he was only marginally below the diagnostic cutoffs for displaying a severe presentation, underscoring the urgent nature of the disease. Infected individuals may quickly deteriorate to a severe status, which may manifest in a variety of ways. Here, our patient displayed concerning features for hematologic and pulmonary involvement. Hematologically speaking, malaria has been reported to cause coagulopathies. *P. falciparum* in particular may result in platelet consumption and prolonged coagulation studies [15], which has previously been summarized [16]. Interestingly though, despite prolonged coagulation studies, malaria is associated with a hypercoagulable state. It has been suggested that this change is involved in the malarial pathogenesis [17]. Additionally, severe malaria has also been reported to manifest with noncardiogenic pulmonary edema [18], even several days after antimalarial therapy initiation. Although the mechanism is unclear, it has been thought to occur due to sequestration of parasitized red cells in pulmonary vasculature or via a cytokine-induced leakage [19]. Importantly, vigorous fluid resuscitation can exacerbate the problem. Our patient presented with poor oral intake and an uptrending creatinine, demanding careful observation of pulmonary status during fluid infusion.

## Conclusions

With only about 2,000 cases in the United States per year, malaria is a rarely encountered illness by most physicians. In an era where Covid represents a likely possibility for flu-like presentations, this case underscores the critical nature of always seeking a thorough social history with inclusion of prior travel. Because patients can rapidly deteriorate to a severe presentation, a prompt diagnosis and treatment initiation with frequent laboratory monitoring is needed for favorable outcomes. Moreover, significant interprofessional collaboration is crucial to maintain high-quality care. Inpatient pharmacy teams and infectious disease should be acutely aware of the patient’s condition, with plans in place to rapidly change medications should the patient deteriorate. Importantly, ACT cannot be used without both infectious disease and pharmacy approval in most institutions within the United States. Discussions should include clear guidance on medication ordering and dosing to prevent treatment delays. Finally, it should be noted that the prevalence of ACT resistance is unknown in the United States, which will require further studies.

## References

[1] (2022). World Health Organization. World malaria report 2021. https://apps.who.int/iris/handle/10665/350147.

[2] Snow RW (2015). Global malaria eradication and the importance of Plasmodium falciparum epidemiology in Africa. BMC Med.

[3] (2022). Centers for Disease Control and Prevention. Malaria: Disease. https://www.cdc.gov/malaria/about/disease.html.

[4] (2022). Centers for Disease Control and Prevention. Malaria: Biology. https://www.cdc.gov/malaria/about/biology/index.html.

[5] Stepniewska K, Humphreys GS, Gonçalves BP (2022). Efficacy of single-dose primaquine with artemisinin combination therapy on Plasmodium falciparum gametocytes and transmission: an individual patient meta-analysis. J Infect Dis.

[6] Collins WE, Jeffery GM (2007). Plasmodium malariae: parasite and disease. Clin Microbiol Rev.

[7] Breman JG (2009). Eradicating malaria. Sci Prog.

[8] (2022). World Health Organization. WHO Guidelines for malaria. https://apps.who.int/iris/handle/10665/351995.

[9] Trampuz A, Jereb M, Muzlovic I, Prabhu RM (2003). Clinical review: severe malaria. Crit Care.

[10] Wilson ME, Weld LH, Boggild A, Keystone JS, Kain KC, von Sonnenburg F, Schwartz E (2007). Fever in returned travelers: results from the GeoSentinel Surveillance Network. Clin Infect Dis.

[11] Svenson JE, MacLean JD, Gyorkos TW, Keystone J (1995). Imported malaria. Clinical presentation and examination of symptomatic travelers. Arch Intern Med.

[12] Nosten F, White NJ (2007). Artemisinin-based combination treatment of Falciparum malaria. Defining and Defeating the Intolerable Burden of Malaria III: Progress and Perspectives.

[13] Gershman MD, Jentes ES, Stoney RJ, Tan KR, Arguin PM (2021). Yellow fever vaccine & malaria prophylaxis information, by country. CDC Yellow Book.

[14] Ashley EA, Dhorda M, Fairhurst RM (2014). Spread of artemisinin resistance in Plasmodium falciparum malaria. N Engl J Med.

[15] Francischetti IM, Seydel KB, Monteiro RQ (2008). Blood coagulation, inflammation, and malaria. Microcirculation.

[16] Bashir BA, Ahmed MS (2020). Laboratory blood coagulation in Sudanese with falciparum malaria: a glance of change outcomes. Int J Blood Res Disord.

[17] Riedl J, Mordmüller B, Koder S (2016). Alterations of blood coagulation in controlled human malaria infection. Malar J.

[18] Taylor WR, Hanson J, Turner GD, White NJ, Dondorp AM (2012). Respiratory manifestations of malaria. Chest.

[19] (2000). Severe falciparum malaria. World Health Organization, Communicable Diseases Cluster. Trans R Soc Trop Med Hyg.

